# Gender Differences in the Psychosocial Determinants Underlying the Onset and Maintenance of Alcohol Use Disorder

**DOI:** 10.3389/fnins.2022.808776

**Published:** 2022-03-14

**Authors:** Andrea M. Maxwell, Katherine Harrison, Eric Rawls, Anna Zilverstand

**Affiliations:** ^1^Medical Scientist Training Program, University of Minnesota, Minneapolis, MN, United States; ^2^Graduate Program in Neuroscience, University of Minnesota, Minneapolis, MN, United States; ^3^Graduate Program in Cognitive Sciences, University of Minnesota, Minneapolis, MN, United States; ^4^Department of Family Medicine and Community Health, University of Minnesota, Minneapolis, MN, United States; ^5^Department of Psychiatry and Behavioral Sciences, University of Minnesota, Minneapolis, MN, United States; ^6^Medical Discovery Team on Addiction, University of Minnesota, Minneapolis, MN, United States

**Keywords:** addiction, gender differences, alcohol use disorder (AUD), social support, causal modeling

## Abstract

A large number of different mechanisms have been linked to Alcohol Use Disorder (AUD), including psychosocial, neurocognitive, affective, and neurobiological factors. Gender has been shown to impact the presentation and progression of AUD; yet, little work has been done to parse the different mechanisms underlying AUD within the lens of gender differences. A review of the literature on adolescence revealed that psychosocial factors, in particular lack of family social support and interactions with peers, drive the onset of alcohol use more strongly in girls relative to boys. However, research done on gender differences in disease progression in adults remains limited. Our gender-specific analysis of the mechanisms underlying AUD in adults revealed that lack of social support was causally linked to negative affect, mental health symptoms, and AUD symptom severity in women, but not men. These novel results suggest that psychosocial factors may play a gender-specific role not only in the onset of use in adolescence, but also in the maintenance of addiction in adults. If confirmed, this suggests the need for investigating gender-specific recovery trajectories. In this perspective piece, we review the literature regarding gender differences in the onset and maintenance of AUD and present original data that support unique risk factors in women.

## Introduction

Alcohol use disorder (AUD) inflicts a huge societal burden and is one of the leading causes of preventable death, with an estimated lifetime prevalence of about 30% (Hasin et al., 2007; Grant et al., 2015). Prevalence rates for AUD are rising among women, with the gender gap narrowing substantially over the past decades (Grant et al., 2017). Despite decades of research that have contributed to understanding the etiology and potential treatments for AUD, relapse rates remain high, and patients report low confidence in the effectiveness of existing treatment options (Grant, 1997; Rapp et al., 2006; Walitzer and Dearing, 2006). Women, in particular, experience more barriers and are less likely to seek treatment, in part because standard treatment approaches have often been developed primarily for men (Vannicelli and Nash, 1984; Brady and Ashley, 2005; Weisner, 2005). Previous research, albeit limited, also evidences gender differences in the onset and pattern of alcohol use (Nolen-Hoeksema, 2004; Becker et al., 2017). Furthermore, escalation from casual use to addiction seems to be more rapid in women than in men, and women are more likely to relapse following a stressful event or drug-related cue relative to men (Becker et al., 2017). The underlying etiology of these gender differences in substance use, however, remains understudied. Biomedical research, in fact, has been optimized to the benefit of men by excluding females in preclinical studies for convenience. It was not until 2016 that the National Institutes of Health mandated that sex as a biological variable be accounted for in preclinical studies (Clayton and Collins, 2014). Thus, prior to this mandate, biomedical research on women in general, including female animal models of addiction, was severely limited. In humans, social, psychological, and neurobiological differences all likely contribute to AUD pathology. To design and implement effective treatments that take gender differences in these factors into account, a thorough understanding of the large number of contributing mechanisms must be obtained. Current neurobiological theories of addiction neglect the role of social factors, despite evidence that social factors may contribute to gender differences in AUD (Becker et al., 2016). Therefore, we will focus on the impact of social factors, specifically social support, on alcohol use and potential gender differences in this relationship. Although a systematic review of the current literature is outside the scope of this perspective piece, we will provide a brief narrative review regarding alcohol use and gender differences in social support among adolescents and adults. We include both subthreshold alcohol use and formal AUD diagnosis in this review because problematic or heavy drinking often precedes a formal AUD diagnosis (Shankman et al., 2009; Beseler et al., 2012; Kranzler and Soyka, 2018). We include literature from both adolescents and adults because adolescent-onset heavy drinking increases the odds of meeting criteria for AUD in adulthood (Yuen et al., 2020). We hence review the influence of social factors on a continuum from adolescence to adulthood, and from heavy drinking to dependence. We also present novel data on the causal mechanisms underlying AUD, and how they differ by gender. These novel data suggest a causal role of social support in AUD in women, but not men.

## Social Support

Social support refers to the extent to which someone perceives their social relationships as able to help them cope in times of stress (Cohen, 2004). The NIH Toolbox Social Relationships Assessment battery synthesizes several scales into a single comprehensive assessment of social support (Cyranowski et al., 2013). This assessment probes six subdomains: emotional support, instrumental support, friendship, loneliness, perceived rejection, and perceived hostility (Cyranowski et al., 2013). Emotional support refers to having someone available who can provide advice and empathy in a difficult situation (e.g., “I feel there are people I can talk to if I am upset”). Instrumental support refers to the availability of individuals who can provide functional aid, such as cooking and shopping (e.g., “Someone is around to make my meals if I am unable to do it myself”). Friendship refers to the existence of friends and companions in one’s social sphere (e.g., “I get invited to go out and do things with other people”). Loneliness refers to self-reported subjective feelings of loneliness (e.g., “I feel alone and apart from others”). Finally, the perceived rejection subdomain probes experiences including experiences of insensitivity or neglect from others (i.e., “people act like my problems aren’t that important”), while perceived hostility probes experiences of a hostile or critical nature as well as active ridicule (“i.e., people blame me when things go wrong”) (Cyranowski et al., 2013). We will briefly review the existing literature regarding each of these subdomains of social support in relation to substance use in adolescents and adults.

## Social Support and Alcohol Use in Adolescents

Several domains of social support are important to the development of substance use in adolescents. Parental support is consistently indicated as a protective factor against the onset and escalation of substance use (for a thorough review see Wills et al., 2014). Parental support, typically defined as a combination of emotional and instrumental support, exerts its protective impact *via* a stress-buffering effect (Wills and Cleary, 1996; Wills et al., 2014). That is, high levels of parental support result in less detrimental impact of a particular stressor on an adolescent. Adolescents between the ages of 9 and 18 with more affirmative parent relationships, defined as those that consist of both emotional closeness and parental supervision, were less likely to have tried alcohol, marijuana, and cigarettes (Tharp and Noonan, 2012). Correspondingly, another study found that in a sample of 9–12th graders, lower levels of family functioning, measured by indices including family problem-solving, affective involvement, and affective responsiveness, increased the magnitude of the association between peer risk behavior and the frequency of marijuana and cigarette use in adolescents (although not heavy episodic drinking), suggesting that lack of family support is a vulnerability factor in adolescent drug use (Miller et al., 1985; Prinstein et al., 2001). In sum, parentally derived emotional and instrumental support protects against the onset and escalation of adolescent substance use, largely by buffering against the impact of negative stressors.

In contrast to the stress-buffering effects typically found for parental support, studies of peer support have indicated that alcohol use is greater in adolescents with high peer support. In fact, one analysis found that parent support and peer support, both measured using a combination of adolescent-reported items probing emotional and instrumental support, had opposite effects on adolescent alcohol use such that parental support was related to fewer symptoms of alcohol use and dependence while peer support was related to more symptoms (Urberg et al., 2005). Moreover, high parental support, even in the context of heavy parental alcohol use, reduced the impact of parental alcohol use on alchol-related symptoms in adolescents. This finding suggests that high parental social support may mitigate the stressful impact of parental alcohol abuse in adolescents (Urberg et al., 2005). Similarly, another study found that in a sample of urban ninth-graders, after controlling for neighborhood and demographic factors, maternal support, measured by both emotional and instrumental support, was associated with less frequent alcohol use among adolescents while peer support, measured by identical emotional and instrumental support items, was related to more frequent alcohol use (Brenner et al., 2011). Thus, high levels of peer support predict alcohol use, in contrast to parental support. This paradoxical finding has been replicated in other studies, and current research proposes that this relationship may be mediated by more risk-taking and higher levels of peer drug use among adolescents with high peer support (Wills et al., 2004). Thus, although the level of support (as measured by the ability to derive sympathy and support from a person) from either a parent or peer may be comparable, divergent values endorsed between parent and peer groups (e.g., peer groups value more risk-taking actions and have more positive attitudes toward alcohol use) may drive different end outcomes (Wills et al., 2004).

Parental rejection, defined as an adolescent’s negative assessment of their emotional connection with a parent, may also be associated with adolescent alcohol use (Stogner and Gibson, 2016). One study of a sample of 7–12th graders reported an association between adolescents who endorsed higher levels of perceived parental rejection (measured by statements referring to the respondent’s satisfaction with the relationship, encouragement from the parent to be independent, good communication, and warmth) and increased alcohol use in those with a genetic vulnerability (Stogner and Gibson, 2016). In reference to perceived parental hostility specifically, the literature appears limited. One recent study found that relative to adolescents who had not reported alcohol use initiation by 8th grade, adolescents who had reported alcohol use initiation by 8th grade were more likely to belong to families reporting high levels of parent-adolescent and parent-parent hostility in 6th grade (Xia et al., 2020). Although parental hostility may also be interpreted as child maltreatment, a review of the child maltreatment literature is outside the scope of this perspective piece. In brief, there is extensive literature regarding the positive association between child maltreatment and adolescent alcohol use (Hamburger et al., 2008; Shin et al., 2009; Cicchetti and Handley, 2019; Hagborg et al., 2020).

Perceived rejection by peers is also associated with alcohol use in adolescents. One study found that chronic peer exclusion at age 12, measured by the Child Social Preference Scale (e.g., “I’d like to hang out with other kids, but I’m often excluded”) was associated with higher levels of alcohol use over the past year at age 19 (Bowker and Raja, 2011; Meisel et al., 2018). When perceived peer hostility is interpreted as peer victimization (i.e., bullying), extant literature supports the association between perceived hostility from peers and adolescent alcohol use, although a recent review notes that conflicting evidence may due to differing sample characterizations and different subtypes of peer victimization (Maniglio, 2017; Meisel et al., 2018).

Research is limited regarding the other subdomains that contribute to social relationships. Loneliness has also been associated with alcohol use in adolescents. One study found that high levels of loneliness in ninth-graders, as measured by a three-item scale probing self-reported feelings of isolation and companionship, was indirectly associated with greater alcohol-related harm (e.g., sick after drinking and trouble with police after drinking) *via* impaired self-efficacy (McBride et al., 2000; McKay et al., 2017). How loneliness and high peer support, two seemingly different ends of the social relationship spectrum, interact to affect alcohol use has yet to be delineated. To our knowledge, there is no research on the relationship between “friendship” as defined by the NIH toolbox and alcohol use in adolescents, although this construct can likely be interpreted in parallel to research regarding peer support.

## Gender Differences in the Relationship Between Social Support and Alcohol Use in Adolescents

Although both familial and peer social support are impactful in the initiation of substance use in both adolescent boys and girls, there appears to be gendered differences in the extent to which these social relationships affect alcohol use in adolescence. For example, one study found in 10- to 15-year-olds, maternal social support buffered the effect of pro-drug use promoting peer affiliation on the frequency and quantity of alcohol use in girls but not boys, such that the association between pro-drug peer affiliation and alcohol use was stronger at lower levels of maternal support relative to high levels of maternal support in girls only (Marshal and Chassin, 2000). Similarly, paternal support buffered the effect of pro-drug use promoting peers on adolescent girls’ alcohol use with no effect on peer influence in boys (Marshal and Chassin, 2000). These findings suggest a differential role of maternal and paternal social support on alcohol use in adolescent girls relative to boys. Furthermore, one study of 12- to 15-year-olds found that family conflict was associated with alcohol and/or marijuana abuse or dependence in girls but not boys, suggesting an elevated vulnerability to AUD within the context of impaired familial social support (Skeer et al., 2011). Similarly, another study of ninth-graders found that higher levels of family social support was related to lower odds of alcohol use in the past month in girls, but not for boys (Nelson et al., 2017), and youth-reported higher quality parent-youth relationships was a stronger protective factor against alcohol initiation in eighth-grade girls relative to boys (Rusby et al., 2018). In sum, these findings suggest that for adolescent girls, familial social support and strong parent-youth relationships seem to be protective against both initiation of alcohol use and alcohol dependence/abuse, but less so in adolescent boys.

There are also gender differences in the relationship between peer-derived social support and alcohol use, although the literature on peer support is mixed. Some research indicates that having peers who drink may be a greater risk to adolescent girls’ drinking behavior relative to boys (Brooks-Russell et al., 2014; Dir et al., 2017). For example, one study of 15- to 19-year-old students reported that girls who reported closer relationships with school-based friends rather than family or church-based friends were more likely to binge drink, while this relationship did not exist in boys (Zarzar et al., 2012). These studies provide insight into the complex role of peer support vs. peer affiliation on alcohol use. For example, one study found in seventh-graders that although the proportion of friends who drink alcohol was predictive of heavy episodic drinking in adolescent girls and boys, strong emotional bonds to peers were protective against heavy episodic drinking in girls only (Danielsson et al., 2011). This finding indicates that further work parsing the effect of peer support (i.e., emotional and instrumental support derived from a peer relationship) vs. peer affiliation (i.e., reported relationship with peers without regard to level of emotional and instrumental support derived from said relationship) in girls relative to boys is necessary. Gender differences in the relationship between perceived rejection and hostility from peers may also exist. For example, one study of 9–12th graders found that although peer victimization in general was predictive of adolescent alcohol use in both boys and girls, the form of peer victimization affected them differently (Kim et al., 2019). The effect of school bulling victimization was predictive of alcohol use in girls, but not boys, while sexual dating violence was predictive of alcohol use in boys, but not girls (Kim et al., 2019). We are not aware of current work on gender differences in the effects of perceived parent rejection.

Furthermore, there is early evidence of gender differences in the other subdomains of social relationships. One recent study reported that in a sample of ninth-graders, girls who reported loneliness were more likely to have had an alcoholic drink in the past month and more likely to ever have consumed a full alcoholic drink, compared to boys who reported experiencing loneliness (McKay et al., 2017). Although this relationship between loneliness and alcohol use should be further explored, these findings provide preliminary evidence that gender differences exist in how loneliness affects future drinking behavior.

## Social Support and Alcohol Use in Adulthood

For both adult men and women, social networks and social support influence substance use behaviors, although the literature remains limited. Previous work indicates that adults with AUD report less family cohesion (e.g., the degree to which family members are helpful and supportive of each other), less expressiveness (e.g., the extent to which family members can express feelings toward each other), and more family conflict (e.g., the degree to which open expressions of anger and aggression are characteristic of the family), as measured by the Family Environment Scale, than adults without AUD (Barry and Fleming, 1990). Similarly, higher levels of work-family conflict, referring to stress between work and family environments (e.g., “Things going on in my family life make it hard for me to concentrate at work”) are associated with increased alcohol use, which is mediated by perceived distress and moderated by high tension reduction expectancies (e.g., “Alcohol makes me worry less”) (Wolff et al., 2013). These findings suggest a relationship between lack of support in managing work-life balance and AUD. Another study measured social support as a combination of tangible (i.e., instrumental) support, appraisal support (i.e., the availability of someone with whom to confide), and belonging support (i.e., the availability of someone with whom to relax), and found that tangible support was negatively related to drinking to cope across men and women (Peirce et al., 1996). Belonging support was negatively related to alcohol problems (e.g., losing a job because of drinking, having blackouts) (Peirce et al., 1996). Moreover, both recovery-specific social support and overall social support were positively associated with increased motivation to reduce alcohol use and motivation to change, respectively, among a sample of individuals with problematic drinking (Moon et al., 2019). Social support from both family and partner, measured by the extent to which the participant felt loved, esteemed by, and involved with others, was also reported as an important factor in maintaining remission following treatment for AUD (Rumpf et al., 2002). This finding is corroborated by a recent study reporting that individuals with AUD whose partners exhibited more negative behaviors (e.g., observer-coded hostility, psychological abuse, and distress-maintaining behaviors while discussing a problem that caused intense disagreement within a laboratory setting) at baseline reported slower declines in drinking trajectories, more frequent drinking, and more alcohol problems over time as well as higher levels of drinking and alcohol-related problems at follow-up (Fairbairn and Cranford, 2016).

Furthermore, although much of the adult literature focuses on family-relevant support, peer relationships, particularly in young adults, may also play a role in alcohol use. One comprehensive review found that a lack or breakdown of quality peer relationships may facilitate alcohol use *via* reduced levels of intimacy, increased alienation, emotional pain, and social support (Borsari and Carey, 2006). For example, in a study of university students, researchers found that students who reported a high number of people who they could rely on for support in stressful times consumed a lower quantity of alcohol during a stressful period (i.e., class examinations) relative to students who reported a lower number of such contacts (Steptoe et al., 1996). Moreover, recent work suggests that time spent with peers and higher perceived friendship, measured by the perceived availability of friends with whom to interact, is related to increased consumption of alcohol among college-aged students (Gesualdo and Pinquart, 2021; Li et al., 2021). The importance of *quality* of peer support vs. *quantity* of peer affiliation may be key in parsing the effect of peer-derived social support on alcohol use in young adults. Taken together, the present literature suggests that social support from both family and peers plays an important role in alcohol use in adulthood.

Previous research regarding loneliness and adult alcohol use is mixed. For example, one review synthesized several different indices of loneliness and its effect on alcohol use, postulating that loneliness facilitates alcohol use at all phase of addiction, including initiation, maintenance, and difficulty in maintaining abstinence (Akerlind and Hörnquist, 1992). This finding is corroborated by a recent study reporting that compared to individuals who reported never feeling lonely, participants who reported loneliness (e.g., “How often have you felt lonely and wished for more friends?”) in the prior 2 weeks exhibited a higher average days of alcohol use over the past month (Gutkind et al., 2022). Similarly, data from the ongoing COVID-19 pandemic provides evidence in young adults linking loneliness to alcohol use such that increasing levels of loneliness, evaluated *via* self-reported feelings of loneliness and social isolation, are related to increased alcohol use severity (as measured by quantity and alcohol-related consequences) *via* heightened anxiety (Horigian et al., 2021). However, an examination of loneliness in adults over the age of 50 found that increased frequency of loneliness was associated with decreased frequency in drinking alcohol, although there was no difference in binge drinking or “at-risk” drinking (characterized as outside of the NIAAA guidelines of “low-risk” drinking) (Canham et al., 2016).

## Gender Differences in the Relationship Between Social Support and Alcohol Use in Adulthood

Literature exploring gender differences in the relationship between social support and alcohol use in adulthood is limited. One study found that neighborhood disorder (e.g., presence of graffiti on buildings, noise nuisance, fear of robbery in the neighborhood) was associated with more hazardous alcohol use (defined as >14 drinks/week for women and >21 for men) for women, but not for men. For men, moderate social cohesion (e.g., feelings of attachment to a neighborhood, being in touch with other neighbors, high levels of neighborhood solidarity) was associated with more hazardous alcohol use (Kuipers et al., 2012). These disparate findings suggest that there may be gender-specific pathways between social support and hazardous alcohol use in adults. Another study found that more welfare contacts, including the receipt of general assistance or welfare (i.e., instrumental support), was related to decreased alcohol consumption in problem-drinking women but not men (Ammon et al., 2008). Interestingly, one intervention designed to alter the social network of individuals with AUD to promote sobriety was more successful in men than in women (Litt et al., 2015). Analysis of this gender difference in treatment outcome found that women with AUD in the treatment group had social systems that were overall less supportive of abstinence than the social circles of their male counterparts (Litt et al., 2015). Thus, women with AUD in this study had stable yet more maladaptive social circles and did not expand their social circles to include sobriety-encouraging members throughout the treatment. A recent study reported that levels of work- family conflict (e.g., “Things I want to do at home and do not get done because of the demands my job puts on me”) was positively associated with increased drinking (both risky single occasion drinking and standard drinks consumed on a drinking day) as a coping mechanism in mothers of young children, but not fathers of young children, suggesting that lack of support managing work-life balance differentially affects alcohol use in mothers relative to fathers (Kuntsche and Kuntsche, 2021). Furthermore, a longitudinal study reported that maternal hostility, measured by observer-coded interactions of mother-toddler dyads at age 2–3.5 years of age, predicted later alcohol use at follow-up at age 19 in the female, but not male children of these mothers (Englund et al., 2008), again suggesting that social interaction has a larger impact on women as compared to men. The field remains limited in work regarding gender differences in the relationship of other subdomains of social support (loneliness, perceived rejection, and perceived hostility) and alcohol use in adults.

## Empirical Data on the Causal Role of Social Support in Alcohol Use Disorder in Women

**FIGURE 1:**
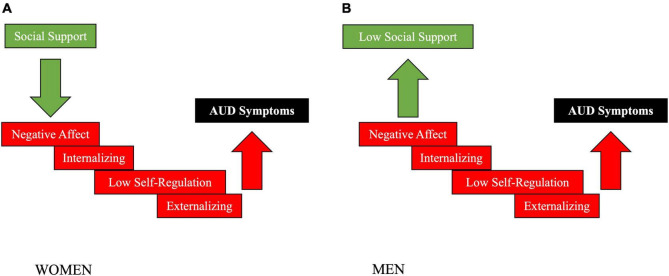
Summary of our empirical gender-specific causal pathway analysis on the role of social support in AUD in women **(A)** vs men **(B)** (see Supplementary Material for details on the approach and results). Causal modeling suggested that increased social support had a buffering effect on negative affect, internalizing and externalizing mental health symptoms, lack of self-regulation and AUD symptom severity in women, but not men. In men, in contrast, negative affect, mental health symptoms and a lack of self-regulation dysregulated both AUD symptom severity and the availability of social support. These novel results support the existing literature by demonstrating that social support plays a larger role in modulating AUD symptom severity in women compared to men.

We have previously used a machine learning method – Causal Discovery Analysis – to analyze the causal factors driving symptom severity in a large sample of adults with AUD (Rawls et al., 2021). This sample was deeply phenotyped through a 2-day assessment that looked at psychological, neurocognitive, affective, neurobiological and social factors. Importantly, our previous analysis, which was not gender-specific, found that social factors played a crucial role in explaining high levels of alcohol use in this sample, as social support buffered the negative effects of impaired cognition, negative affect and mental health issues that were causally linked to AUD symptom severity (Rawls et al., 2021). To investigate if there is a gender-specific role of social support as suggested by our literature report, we conducted a gender-specific CDA within the same sample. The methods and results for this analysis are described in detail in the Supplementary Material, and the main result of this analysis is summarized in Figure 1. In short, we found that in women, increased social support had a buffering effect on negative affect, internalizing and externalizing mental health symptoms, and lack of self-regulation, which in turn improved AUD symptom severity. In men, there was no such effect; instead, negative affect, mental health symptoms, and lack of self-regulation dysregulated both AUD symptom severity and the availability of social support. Our gender-specific causal pathway analysis thus converged with the literature to demonstrate that social support plays a larger role in modulating AUD symptom severity in women as compared to men. Based on both our literature review and the provided novel empirical results, we conclude that social factors play a larger role as a causal mechanism underlying problematic drinking and alcohol dependence in women than in men.

## Perspective

Our novel empirical findings confirm the reviewed literature and solidify the conclusion that social support plays a unique role in the maintenance of AUD in women as compared to men. While many of the complexities in the details remain to be researched, our literature review did reveal a remarkable continuity from adolescence into adulthood regarding the importance of social relationships in girls and women, both as a risk and protective factor. Equally, our empirical data analysis supported the importance of social support in maintaining problematic alcohol use and alcohol dependence in women, but not men. However, parsing specific aspects of social support, and which aspects are most targetable by interventions, has yet to be done.

Furthermore, exciting novel work in preclinical models supports the role of social influences on alcohol drinking (Heilig et al., 2016). A growing area of work has extended this research to probe the effect of sex on alcohol drinking in animal models. For example, in one animal model of adolescent peer rejection, peer-rejected female rats exhibited increased alcohol-seeking behavior following extinction relative to control females, while males exhibited reduced alcohol-seeking behavior after peer-rejection (Surakka et al., 2021). Additionally, the effect of social interaction on adolescent drinking behavior has been modeled in rats using a variety of paradigms, including the demonstrator-observer paradigm. In this paradigm, the demonstrator animal is exposed to ethanol, and a change in social behavior as well as voluntary ethanol consumption is measured in the observer animals. One recent study using this paradigm found that only female adolescent rats exposed to ethanol-intoxicated peers exhibited increased voluntary ethanol consumption, suggesting sex-specific differences in peer influence on alcohol intake (Maldonado et al., 2008; Gamble et al., 2019).

These studies, among others, corroborate human work indicating gender-specific effects of social support on alcohol use. As the gender gap in AUD is closing, research provides alarming early evidence of a more rapid escalation of substance use and increased barriers to treatment in women. It will be vital to the wellbeing of women to understand the role of the social environment and related targetable treatment approaches. For example, interventions focused on promoting peer-supported sobriety, and effectively utilizing emotional support from family and health care providers, may be more tailored toward successful remission in women. Furthermore, large-scale interventions targeting the development and distribution of instrumental support from governmental agencies and treatment providers may be more successful in addiction treatment in women. Combining this knowledge of the impact of social systems on addiction with further research on the role of hormonal cycles, cognitive processing, and brain structure in women may provide treatment providers the tools to best conceptualize and target precise, individualized treatment plans for their patients. Furthermore, in addition to benefiting women themselves, women remain the primary caretakers of children in the United Sates. Providing effective treatment for parenting and pregnant women will inherently provide children and their families with better tools for a healthier future (Women’s Bureau, U.S. Department of Labor, 2019).

## Data Availability Statement

Publicly available datasets were analyzed in this study. This data can be found here: https://www.humanconnectome.org/study/hcp-young-adult/data-releases.

## Ethics Statement

The studies involving human participants were reviewed and approved by the Washington University Institutional Review Board. The patients/participants provided their written informed consent to participate in this study.

## Author Contributions

AM conducted the literature review and empirical analysis and drafted the manuscript. KH conducted the literature review and provided critical edits. ER conducted the empirical analysis and provided critical edits. AZ conceptualized and funded the project, provided supervision and critical edits to all sections of the manuscript. All authors contributed to the article and approved the submitted version.

## Conflict of Interest

The authors declare that the research was conducted in the absence of any commercial or financial relationships that could be construed as a potential conflict of interest.

## Publisher’s Note

All claims expressed in this article are solely those of the authors and do not necessarily represent those of their affiliated organizations, or those of the publisher, the editors and the reviewers. Any product that may be evaluated in this article, or claim that may be made by its manufacturer, is not guaranteed or endorsed by the publisher.
