# Supplementary material for: Gender Differences in the Psychosocial Determinants Underlying the Onset and Maintenance of Alcohol Use Disorder
Source: Front Neurosci. 2022 Mar 14;16:808776. doi: 10.3389/fnins.2022.808776 (PMC8964095; doi:10.3389/fnins.2022.808776)
Supplement: Supplementary file 1 [file Data_Sheet_1.pdf]

## SUPPLEMENTARY MATERIALS for:

# Gender differences in the psychosocial determinants underlying the onset and maintenance of Alcohol Use Disorder

## Supplementary Analysis:

## An empirical analysis of the causal role of social support in Alcohol Use Disorder in women versus men

### Introduction

A multi-causality of mechanisms has been linked to Alcohol Use Disorder (AUD), including psychosocial, neurocognitive, affective, and neurobiological factors (Rawls, Kummerfeld, and Zilverstand 2021). Previously, we applied a data-driven machine learning technique to derive causal graphs that encompassed brain-derived, cognitive, social, and affective factors contributing to AUD severity (Rawls, Kummerfeld, and Zilverstand 2021). To do so, we used a community sample of healthy young adults and employed an exploratory factor analysis (EFA) to parse 100 phenotypic measures into 18 latent factors. We then input these 18 latent factors into a Causal Discovery Analysis (CDA), a class of data-driven machine learning approaches that models causal relationships between factors. In brief, we found that causal influence propagated from brain-derived factors (i.e., brain connectivity) to cognitive factors (e.g., working memory, crystalized IQ), to social factors (e.g., social support), and finally to affective and psychiatric function (e.g., negative affect, internalizing & externalizing symptoms), which was directly causally linked to AUD symptom severity (Rawls, Kummerfeld, and Zilverstand 2021).

In this prior analysis, however, we did not take gender into account, despite evidence that gender has been shown to impact the presentation and progression of AUD. Thus, in the current study, we extend this previously published analysis and perform a separate CDA for men and women to test whether casual graphs of AUD severity differ by gender.

### Methods

**Subjects**. We analyzed data from the Human Connectome Project (HCP; *N* = 1206, aged 22-35, 54% females) (Van Essen et al. 2013). All subjects provided written informed consent at Washington University. The Causal Discovery Analysis included all participants with complete phenotypic (*N* = 933) and resting-state fMRI (*N* = 1085), with a final sample of *N* = 926 (37% female). Of the final sample, 204 individuals (22%) of the final sample met criteria for AUD (37% of which were women).

**Table 1. Demographics of Final Sample**

| Demographics | AUD Women *N* = 76 (15% of women) | AUD Men *N* = 128 (30% of men) | HC Women *N* = 422 | HC Men *N* = 300 | Total *N* = 926 |
|---|---|---|---|---|---|
| White | 58 | 108[a] | 311 | 223[a] | 700 |
| Black / AA | 7 | 8[a] | 72 | 43[a] | 130 |
| Asian | 4 | 4 | 26[b] | 23[b] | 57 |
| First Nations | 0 | 0 | 1 | 1 | 2 |
| Other | 7 | 8 | 12 | 10 | 37 |
| Age Mean (SD) | 28.48(3.02)[c] | 28.80 (3.57)[c] | 29.72(3.61)[c] | 27.70 (3.6)[c] | 28.83(3.67) |
| Income Mean (SD) | 4.92 (2.17) | 5.02 (2.16) | 5.20 (2.12) | 5.02 (2.14) | 5.10(2.13) |
| Education Mean (SD) [d] | 14.91 (1.88) | 14.98 (1.66) | 15.12 (1.75)[c] | 14.81 (1.79)[c] | 14.98 (1.77) |

AUD = Alcohol Use Disorder; HC = Healthy Control; AA= African American; SD= Standard Deviation

[a] Effect of AUD, $p < 0.05$, calculated using Pearson's Chi-Squared Test

[b] Effect of gender, $p < 0.05$, calculated using Pearson's Chi-Squared Test

[c] Effect of gender, $p < 0.05$, calculated using Welch Two Sample T-Test

[d] Income was binned (1 = <10K, 2 = 1,1K-19,999, 3 = 20K-29,999, 4 = 30K-39,999, 5 = 40K-49,999, 6 = 50K-74,999, 7 = 75K-99,999, 8 = ≥100K).

**AUD symptom severity.** The HCP dataset assessed symptoms of alcohol abuse and dependence based on DSM-IV-TR criteria using the Semi-Structured Assessment for the Genetics of Alcoholism (SSAGA). Because the DSM-V now characterizes alcohol abuse and dependence as a single Alcohol Use Disorder (AUD), we combined the symptom counts of alcohol abuse and dependence into one AUD symptom severity outcome. Because we were interested in both problematic drinking and subthreshold drinking as well as a formalized diagnosis, we used the AUD symptom count as the primary outcome variable.

**Behavioral measures:** Rawls et al. conducted an exploratory factor analysis of all available phenotypic HCP behavioral and self-report measures. In this analysis, 18 latent factors were identified:

> *"Factors, in order of common variance accounted for, were associated with: (1) Somaticism (high DSM/ASR somaticism, high DSM depression, low PSQI sleep quality), (2) Fluid Cognition (high Raven's progressive matrices performance), (3) Internalizing (high DSM/ASR anxiety, high DSM depression, high NEO-FFI neuroticism), (4) Gambling Task Reaction Time (slow gambling task reaction time), (5) Conscientiousness/ Attention (low DSM attention deficit hyperactivity disorder, low ASR attention problems, and high NEO-FFI conscientiousness), (6) Visuospatial Processing (high Penn short line orientation task performance), (7) Social Support (high NIH toolbox friendship, low loneliness, low perceived rejection and perceived hostility, high emotional and instrumental support), (8) Processing Speed (high NIH Toolbox Flanker Total Score, fast fMRI emotion task RT), (9) Externalizing (high ASR aggression and rule-breaking, high DSM antisocial, high NIH toolbox aggression), (10) Social Withdrawal (high ASR withdrawal, high DSM avoidance, low NEO-FFI extraversion), (11) Language Task Performance (high fMRI language task story average difficulty, and high math problem accuracy), (12) Relational Task Reaction Time (slow fMRI relational task reaction time [RT]), (13) Delay Discounting (high delay discounting AUC for $200 and $40k), (14) Working memory (fMRI N-Back task fast reaction time [RT] and high accuracy), (15) Negative Affect (high NIH toolbox anger, fear, sadness and stress), (16) Crystalized IQ (high NIH toolbox English reading and picture vocabulary, high education, and high NEO-FFI openness), (17) Positive Affect (high NIH toolbox life satisfaction, positive affect, and meaning and purpose, and NEO-FFI extraversion), and (18) Agreeableness (low aggression and high NEO-FFI agreeableness)."* (Rawls, Kummerfeld, and Zilverstand 2021).

Of note, the term ‘conscientiousness’ is a trait included in the Big Five taxonomy of personality traits and refers to the tendency of one to behave according to socially acceptable norms, to engage in goal-directed behavior, to plan, and to delay gratification (Roberts et al. 2014). For clarity, we refer to ‘conscientiousness’ in our conceptual framework (**Figure 1**) with the general term ‘self-regulation’. Low levels of conscientiousness have been associated with substance use disorders, as well as low levels of self-regulation (Volkow, Michaelides, and Baler 2019; Ruiz, Pincus, and Schinka 2008; Anderson et al. 2007). Furthermore, ‘internalizing’ refers to maladaptive behaviors and emotional states that are turned ‘inward’ on oneself, such as anxiety and low mood (Hussong et al. 2011). In contrast, externalizing behaviors are behaviors that are turned ‘outward’ to others, such as aggression and rule-breaking (Frick et al. 1993; Bongers et al. 2008). Both internalizing and externalizing behaviors are mental health symptoms that, when impairing, contribute to mental health diagnoses, such as Generalized Anxiety Disorder or Substance Use Disorder, among other diagnoses (Higa-McMillan et al. 2008; Winters et al. 2008) . The construct ‘negative affect’ is a general reference to aversive mood states, such as anger, fear, and nervousness, and is a well-described contributor to substance use disorders (Stasiewicz and Maisto 1993; Watson, Clark, and Tellegen 1988).

For our current analysis, these 18 latent factors were used as inputs into a separate CDA for men and women.

**CDA: Greedy Fast Causal Inference.** Causal models indicate cause-and-effect relationships within a dataset (Pearl 2000). Because datasets with a large number of variables inherently also have a large number of potential causes-and-effect relationships, identification of the model that best fits the data can be challenging. Causal Discovery Analysis (CDA) is a machine learning technique that allows for identification of which causal model within this vast array of possibilities best fit the data (Spirtes, Glymour, and Scheines 2000). Following the same procedures as in Rawls and colleagues (2021), we used Greedy Fast Casual Inference (GFCI), an algorithm that can establish causal relationships even in the presence of unmeasured confounds (Ogarrio, Spirtes, and Ramsey 2016). This property of GFCI makes it well-poised for assessing large samples of human data, where all potential confounds cannot possibly be identified and accounted for. GFCI was conducted in Tetrad using default parameters (alpha = 0.01, maximum degree of the graph = 100, penalty discount = 2). The factor ‘Fluid Cognition’ was added to the Knowledge Box in Tetrad such that no other factors could cause Fluid Cognition, since this factor was at the top of the hierarchy in the full sample that included both men and women (Rawls, Kummerfeld, and Zilverstand 2021). Bayesian Information Criteria was used to calculate the penalized likelihoods for the models, which is the default in Tetrad and a common model fit index in CDA (Schwartz 1978). One CDA was conducted with the sample restricted to only men. A separate CDA was conducted with the sample restricted to only women. The two resultant graphs were visually compared to interpret gender-specific pathways.

## Results

The discovered causal graphs were in large part similar between men and women, see Supplementary Figure 1 for a detailed description of these pathways. Crucially, however, we found that a lack of social support was causally linked to AUD symptom severity in women, but not men. Specifically, low social support was causally linked to AUD symptom severity in women because it was a cause of negative affect, which in turn caused internalizing, low conscientiousness, high externalizing, and finally AUD symptom severity. In men, however, negative affect lead to lower social support, which was causally linked to low positive affect and high social withdrawal, neither of which lead to further constructs that caused overall AUD symptoms.

## Discussion

In summary, our gender-specific pathway analysis indicated that social support was linked to AUD symptom severity in women but not men. Based on these results, we conclude that in women, social support protects against increasing AUD symptom severity by decreasing levels of negative emotionality (i.e., negative affect, internalizing, and externalizing symptoms) and increasing conscientiousness. In contrast, in men, social support does not serve as a buffer of negative emotionality. Instead, negative emotionality (i.e., negative affect), caused lower social support. Low social support was not directly or indirectly linked to AUD symptom severity in men.

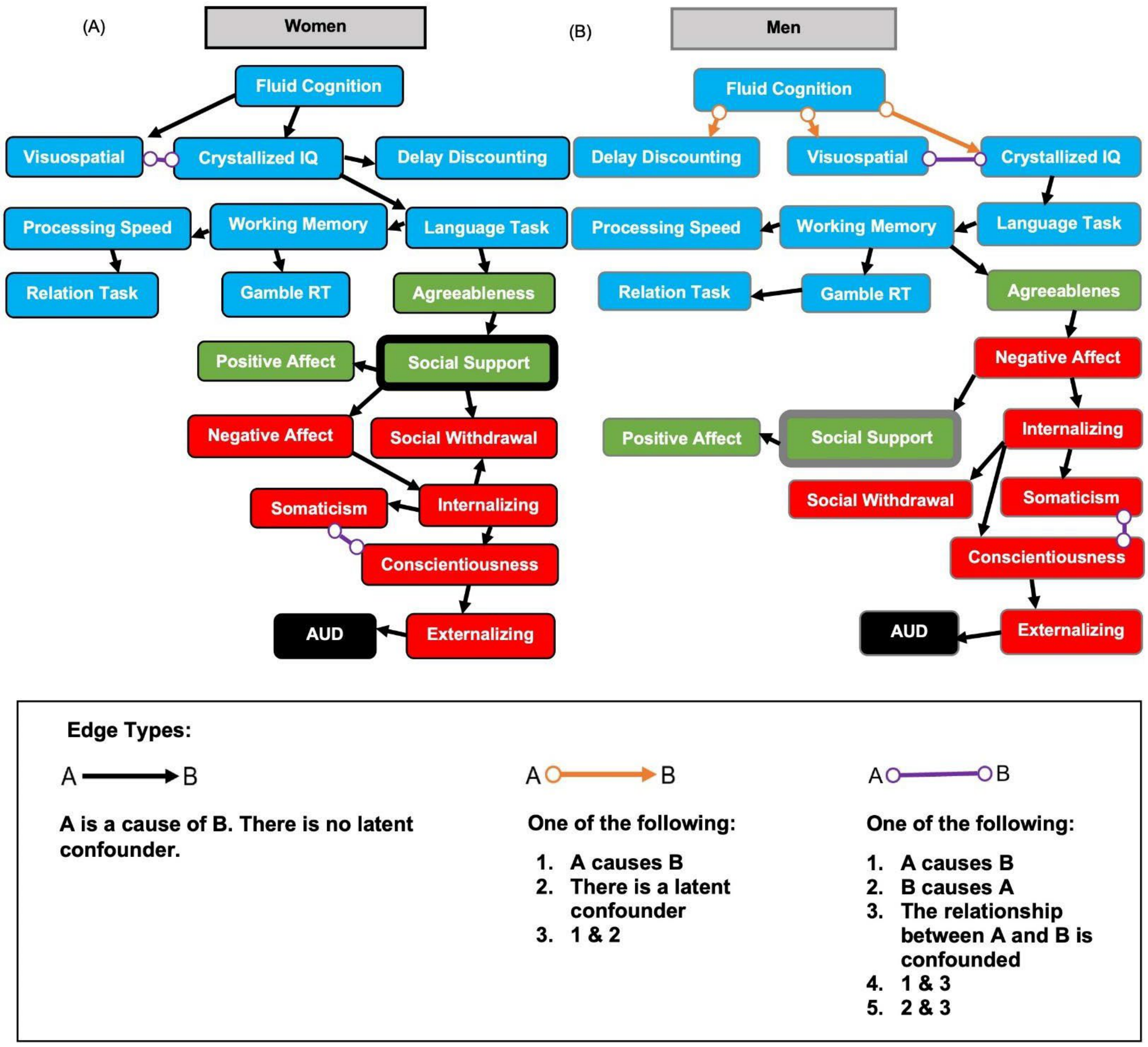


**Supplementary Figure 1**: Causal discovery of the factors contributing to Alcohol Use Disorder symptom severity in women (A) and men (B). The CDA method is a data-driven machine learning approach to modeling causal relationships, which searches an enormous set of possible structural models and returns a graph representing estimated causal relationships in the data. In women, low social support caused negative affect,

which in turn cascaded to high internalizing, low conscientiousness, and high externalizing, which caused AUD symptom severity (A). In men, low social support caused low positive affect and high social withdrawal, but these did not cause AUD symptom severity (B). Thus, low social support was a cause of AUD symptoms in women, but not men. The edge types identify three potential ways in which variables can be causally related. The black arrow indicates that A is a cause of B, without a potential unmeasured confounder variable. The orange arrow indicates that (1) A may cause B, (2), there exists an unmeasured confounder in this relationship, or (3) both 1 & 2. The purple edge indicates that (1) A causes B OR (2) B causes A OR (3) there is an unmeasured confounder in this relationship OR (4) 1 & 3 OR (5) 2 & 3. Factors are color-coded according to domain. Blue = Cognition, Green = Social, Red = Negative Valence.

## References


Anderson, Kristen G., Susan F. Tapert, Ida Moadab, Thomas J. Crowley, and Sandra A. Brown. 2007. "Personality Risk Profile for Conduct Disorder and Substance Use Disorders in Youth." *Addictive Behaviors* 32 (10): 2377–82. https://doi.org/10.1016/j.addbeh.2007.02.006.

Bongers, I. L., H. M. Koot, J. van der Ende, and F. C. Verhulst. 2008. "Predicting Young Adult Social Functioning from Developmental Trajectories of Externalizing Behaviour." *Psychological Medicine* 38 (7): 989–99. https://doi.org/10.1017/S0033291707002309.

Frick, Paul J., Benjamin B. Lahey, Rolf Loeber, Lynne Tannenbaum, Yolanda Van Horn, Mary Anne G. Christ, Elizabeth A. Hart, and Kelly Hanson. 1993. "Oppositional Defiant Disorder and Conduct Disorder: A Meta-Analytic Review of Factor Analyses and Cross-Validation in a Clinic Sample." *Clinical Psychology Review* 13 (4): 319–40. https://doi.org/10.1016/0272-7358(93)90016-F.

Higa-McMillan, Charmaine K., Rita L. Smith, Bruce F. Chorpita, and Kentaro Hayashi. 2008. "Common and Unique Factors Associated with DSM-IV-TR Internalizing Disorders in Children." *Journal of Abnormal Child Psychology* 36 (8): 1279–88. https://doi.org/10.1007/s10802-008-9250-8.

Hussong, Andrea M., Deborah J. Jones, Gabriela L. Stein, Donald H. Baucom, and Sara Boeding. 2011. "An Internalizing Pathway to Alcohol Use and Disorder." *Psychology of Addictive Behaviors* 25 (3): 390–404. https://doi.org/10.1037/a0024519.

Ogarrio, Juan Miguel, Peter Spirtes, and Joe Ramsey. 2016. "A Hybrid Causal Search Algorithm for Latent Variable Models." *JMLR Workshop and Conference Proceedings* 52 (August): 368–79.

Pearl, J. 2000. *Causality: Models, Reasoning, and Inference.* Cambridge University Press.

Rawls, Eric, Erich Kummerfeld, and Anna Zilverstand. 2021. "An Integrated Multimodal Model of Alcohol Use Disorder Generated by Data-Driven Causal Discovery Analysis." *Communications Biology* 4 (1): 435. https://doi.org/10.1038/s42003-021-01955-z.

Roberts, Brent W., Carl Lejuez, Robert F. Krueger, Jessica M. Richards, and Patrick L. Hill. 2014. "What Is Conscientiousness and How Can It Be Assessed?" *Developmental Psychology* 50 (5): 1315–30. https://doi.org/10.1037/a0031109.

Ruiz, Mark A., Aaron L. Pincus, and John A. Schinka. 2008. "Externalizing Pathology and the Five-Factor Model: A Meta-Analysis of Personality Traits Associated with Antisocial Personality Disorder, Substance Use Disorder, and Their Co-Occurrence." *Journal of Personality Disorders* 22 (4): 365–88. https://doi.org/10.1521/pedi.2008.22.4.365.

Schwartz, G. 1978. "Estimating the Dimension of a Model." *Annals of Statistics* 6: 461–64.

Spirtes, P., C.N. Glymour, and R. Scheines. 2000. *Causation, Prediction, and Search*. MIT Press.

Stasiewicz, Paul R., and Stephen A. Maisto. 1993. "Two-Factor Avoidance Theory: The Role of Negative Affect in the Maintenance of Substance Use and Substance Use Disorder." *Behavior Therapy* 24 (3): 337–56. https://doi.org/10.1016/S0005-7894(05)80210-2.

Van Essen, David C., Stephen M. Smith, Deanna M. Barch, Timothy E.J. Behrens, Essa Yacoub, and Kamil Ugurbil. 2013. "The WU-Minn Human Connectome Project: An Overview." *NeuroImage* 80 (October): 62–79. https://doi.org/10.1016/j.neuroimage.2013.05.041.

Volkow, Nora D., Michael Michaelides, and Ruben Baler. 2019. "The Neuroscience of Drug Reward and Addiction." *Physiological Reviews* 99 (4): 2115–40. https://doi.org/10.1152/physrev.00014.2018.

Watson, David, Lee Anna Clark, and Auke Tellegen. 1988. "Development and Validation of Brief Measures of Positive and Negative Affect: The PANAS Scales." *Journal of Personality and Social Psychology* 54 (6): 1063–70. https://doi.org/10.1037/0022-3514.54.6.1063.

Winters, Ken C., Randy D. Stinchfield, William W. Latimer, and Andrea Stone. 2008. "Internalizing and Externalizing Behaviors and Their Association with the Treatment of Adolescents with Substance Use Disorder." *Journal of Substance Abuse Treatment* 35 (3): 269–78. https://doi.org/10.1016/j.jsat.2007.11.002.
